# The effects of medications for treating COPD and allied conditions on stroke: a population-based cohort study

**DOI:** 10.1038/s41533-021-00267-3

**Published:** 2022-01-17

**Authors:** Ai-Ling Shen, Hsiu-Li Lin, Hsiu-Chen Lin, Jane Chen-Jui Chao, Chien-Yeh Hsu, Chung-Yu Chen

**Affiliations:** 1 Department of Neurology, Sijhih Cathay General Hospital, New Taipei City, Taiwan; 2 Department of Pediatrics, School of Medicine, College of Medicine, Taipei Medical University, Taipei, Taiwan; 3 Department of Laboratory Medicine, Taipei Medical University Hospital, Taipei, Taiwan; 4 School of Nutrition and Health Sciences, College of Nutrition, Taipei Medical University, Taipei, Taiwan; 5 Nutrition Research Center, Taipei Medical University Hospital, Taipei, Taiwan; 6 Department of Information Management, National Taipei University of Nursing and Health Sciences, Taipei, Taiwan; 7 Department of Internal Medicine, National Taiwan University Hospital Yunlin Branch, Yunlin County, Taiwan; 8 Division of Pulmonary and Critical Care Medicine, Department of Internal Medicine, National Taiwan University Hospital, College of Medicine, National Taiwan University, Taipei, Taiwan

**Keywords:** Therapeutics, Chronic obstructive pulmonary disease

## Abstract

Patients with chronic obstructive pulmonary disease (COPD) are at higher risk of stroke. This study aimed to investigate the clinical factors of stroke risk in COPD and allied conditions patients and associations between medications for treating COPD and allied conditions. The population-based study cohort comprised 24,173 patients diagnosed with COPD and allied conditions between 2000 and 2013, and 24,170 selected matched patients without COPD comprised the comparison cohort from a nationwide database. Cox-proportional hazard regression was performed to determine the impact of medical therapies, comorbidities, and other clinical factors on stroke risk. Of the 48,343 included patients, 1394 (2.9%) experienced stroke during follow-up, with a significant difference between COPD and allied conditions cohort (1003/4.2%) and comparison cohort (391/1.6%) (adjusted hazard ratio [aHR]: 2.72, *p* < 0.001). Cox-regression analysis revealed that COPD and allied conditions patients who were older (>65 years) (HR: 1.06); male (HR: 1.39); with hypertension (HR: 1.46), diabetes mellitus (HR: 1.33) and atrial fibrillation (HR: 1.63) had increased stroke risk. Mucolytics (HR: 0.44) and combination therapy with inhaled corticosteroids (ICS) and long-acting β2-agonists (LABA) (HR: 0.75) were associated with decreased stroke risk in COPD and allied conditions patients. Among COPD and allied conditions patients, major comorbidities increase risk of stroke. Therapy with mucolytic agents and combination ICS/LABA is associated with risk reduction.

## Introduction

Chronic Obstructive Pulmonary Disease (COPD) is the third leading cause of death worldwide^1^. COPD is a complex respiratory disorder characterized by chronic air flow limitations and increased inflammatory response in the airways. The prevalence of comorbidities is also reported to be higher in patients with COPD than in non-COPD patients and have a significant impact on prognosis^1,2^. The most prevalent comorbidities include cardiovascular disease (hypertension, ischaemic heart disease, heart failure), metabolic syndrome (including obesity, diabetes mellitus, hyperlipidemia), and chronic kidney disease (CKD)^1–3^. COPD in the presence of these comorbidities is considered a risk factor associated with stroke^4,5^. Many population-based studies have found associations between COPD and stroke^6–12^ and increased evidence from recent systematic reviews and meta-analyses have indicated that stroke risk is higher among COPD patients than in non-COPD patients^13–15^. Potential mechanisms by which COPD and stroke may be linked include systemic inflammation, hypoxia, hypercapnia, and oxidative stress^16,17^.

Inhaled bronchodilators, including long-acting β2-agonists (LABA) and long-acting muscarinic antagonists (LAMA), and inhaled corticosteroids (ICS) are cornerstone therapies for COPD patients^1^. The clinical efficacy of inhaled bronchodilators has been demonstrated in clinical trials^18^, including improvements in overall quality of life, prevention of deteriorating lung function, and reducing the frequency of acute exacerbations leading to hospitalization. However, several studies have raised concerns that inhaled bronchodilators increase the risk of cardiovascular events^18–24^. Therefore, the safety of COPD medications is still being debated.

The present study sought to further investigate the relationship between COPD and incident stroke in COPD patients compared to the general population in Taiwan. The primary aim of this study was to clarify whether COPD and allied conditions patients are at increased incidence of stroke over time. Secondary aims were to identify the risk factors for stroke among COPD and allied conditions patients and associations between medications for treating COPD and allied conditions and stroke.

## Methods

### Data source

This study extracted patient data from the Longitudinal Health Insurance Database (LHID), which is a random subset of the Taiwan National Health Insurance Research Database (NHIRD) and contains all medical claims data from one million patients insured during 2000 through 2013. The NHIRD was established in 1996 and encompasses all claims data of medical services used by nearly 99% of 23 million Taiwanese. No significant differences are shown in the distribution of age and sex between the LHID subset and the original NHIRD. All data related to personal identification were encrypted by the National Health Insurance Administration (NHIA) before being published. The confidentiality of patients in the dataset is protected by NHIA data regulations.

All the information related to personal identification had been encrypted by the Bureau of National Health Insurance of Taiwan before the dataset was published. The confidentiality of patients in the dataset was protected. Therefore, this study was exempt from full review by the Institutional Review Board of Sijhih Cathay General Hospital. All study procedures have been performed in accordance with the ethical standards laid down in the 1964 Declaration of Helsinki and its later amendments.

### Study sample

**Fig. 1:**
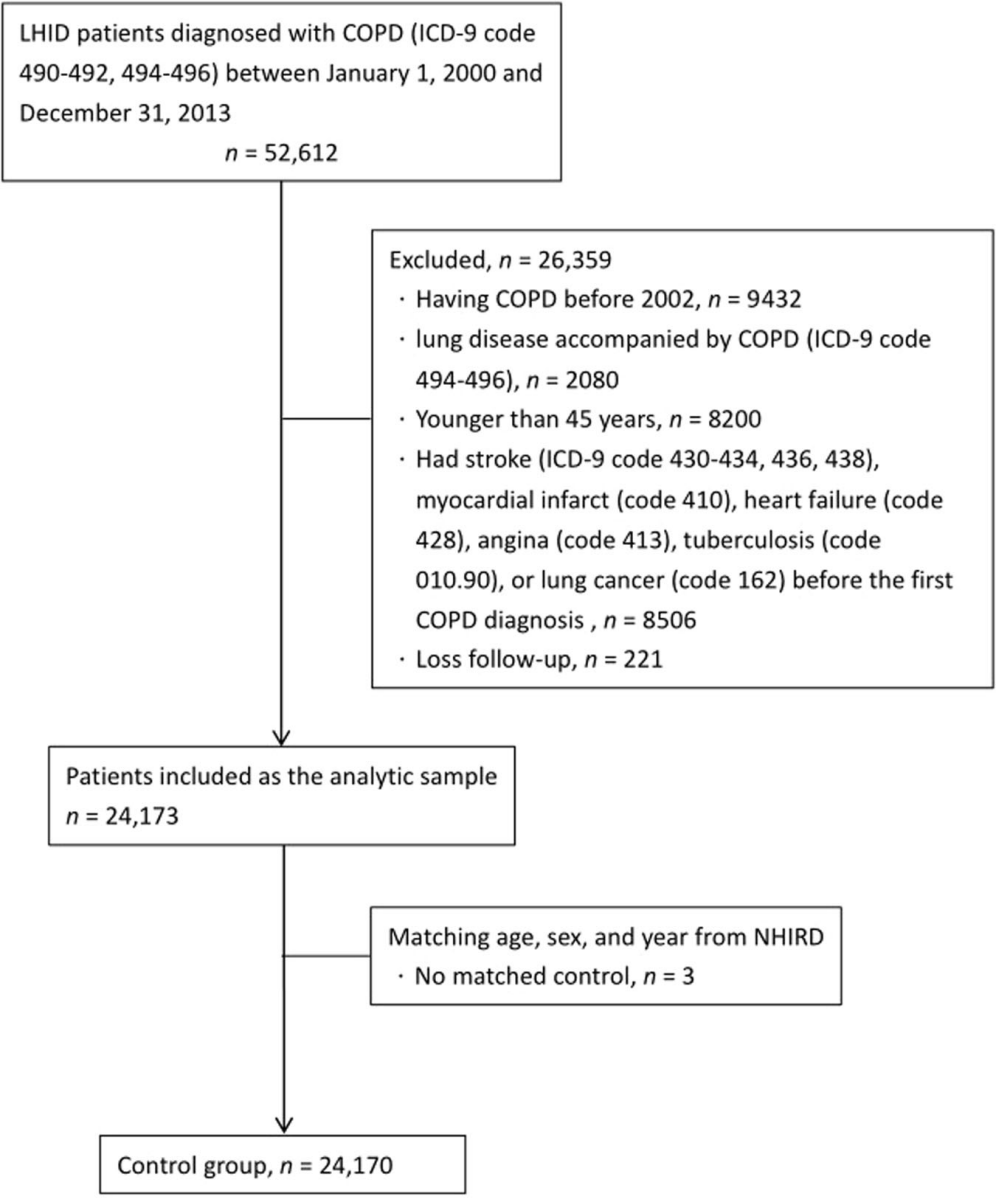
Flow chart of the study population. LHID Longitudinal Health Insurance Database, COPD Chronic obstructive pulmonary disease, NHIRD National Health Insurance Research Database.

We identified all LHID patients who were diagnosed with COPD and allied conditions (ICD-9 code 490–493 [490 Bronchitis], not specified as acute or chronic; 491 Chronic bronchitis; 492 Emphysema; 493 Asthma) by pulmonary specialists in outpatient services and hospital admission records between January 1, 2000, and December 31, 2013 (*n* = 52,612). Because this study aimed to assess the risk of stroke in the newly-diagnosed COPD and allied conditions population, patients with any record of having COPD and allied conditions before 2002 (*n* = 9432) were excluded. We excluded code 494–496 patients with lung disease accompanied by COPD (494 Bronchiectasis; 495 Extrinsic allergic alveolitis; 496 Chronic airway obstruction, not elsewhere classified) (*n* = 2080). We also excluded those who were younger than 45 years (*n* = 8200) and those who had stroke (ICD-9 code 430–434, 436, 438), myocardial infarct (code 410), heart failure (code 428), angina (code 413), tuberculosis (code 010.90), or lung cancer (code 162) before the first COPD diagnosis (*n* = 8506). Patients who had no further follow-up in their medical records after admission for COPD and allied conditions, which may be suggestive of death, were also excluded (*n* = 221). After all exclusions, 24,173 patients were included as the analytic sample (Fig. 1).

To construct the control group, for each included patient, one non-COPD and allied conditions patient was randomly selected from the database by matching age, sex, and year of enrolment. The same exclusion criteria for the COPD and allied conditions group were applied to selection of controls. Three patients had no matched control, therefore, the control group included only 24,170 subjects.

In the process of selecting control group, we firstly excluded patients with diagnosis of COPD and allied conditions (*n* = 52,612) from the one million patients of original Longitudinal Health Insurance Database. We extracted all the out-patient visit records between 2002 and 2013 of every candidate for control group. We divided these records into 24 groups based on the year of visit date and sex of patient. Because one patient may have multiple visit records during a year, we randomly select one index record for each candidate in each year. Next, we divided COPD and allied conditions patients into 24 groups based on the year of the earliest date of COPD and allied conditions diagnosis and sex of patient. For each COPD and allied conditions patient, we randomly selected one age-matched patient in the corresponding sex and year group, and excluded this control patient from the following matching process to assure no duplicate selection.

### Study design

For the studied cases, the index date was defined as the earliest date of COPD and allied conditions diagnosis. For the control group without COPD and allied conditions, the first outpatient department visit date in the comparison year was assigned as the index date. Each patient in both groups was tracked to the earliest date admitted to hospital with a principal diagnosis of stroke (ICD-9 code 430–434, [430 Subarachnoid haemorrhage, 431 Intracerebral haemorrhage, 432 Other and unspecified intracranial haemorrhage, 433 Occlusion and stenosis of precerebral arteries, 434 Occlusion of cerebral arteries], and 436 Acute, but ill-defined, cerebrovascular disease) or to the last outpatient record date in the database if no stroke was diagnosed. The period between the index date and the end tracking date was recorded for additional survival analyses. The demographic data, including date of birth and sex, were extracted from the claims data.

Four related comorbidities were retrieved: hypertension (ICD-9 code 401–405), diabetes mellitus (DM, code 250), dyslipidemia (code 272), and atrial fibrillation (AF, code 427.31) to evaluate the influence of these comorbidities on stroke incidence during the study period.

### Main measures

The hazard ratios (HRs) for the time-to-event model for stroke was compared between patients with COPD and allied conditions (COPD group) and without COPD and allied conditions (non-COPD control group). To evaluate risk factors for stroke among COPD and allied conditions patients, prescription days were summed for each class of COPD and allied conditions medications received during the entire observation period according to the Anatomical Therapeutic Chemical Classification System with Defined Daily Doses (ATC/DDD)^25^. The classes of interest drugs approved in Taiwan include short-acting inhaled beta-agonists (SABA, ATC code R03AC02), long-acting inhaled beta-agonists (LABA, R03AC12, 13, 18, 19), short-acting muscarinic antagonists (SAMA, R03BB01), long-acting muscarinic antagonists (LAMA, R03BB04, 06, 07), LAMA combined with LABA (R03AL03, 06), methylxanthines (R03DA04), mucolytics (R05CB01), inhaled glucocorticosteroids (ICS, R03BA01, 02, 05, 08), and ICS combined with LABA (R03AK06, 07, 10). “Total prescription days” were defined as more than 60 days for users of the specific medication class. HRs was calculated for each demographic factor, comorbidities, and drug classes for stroke among COPD and allied conditions patients. The recurrence of stroke (re-stroke) was defined as another hospital admission with the principle diagnosis of stroke three months later after the index stroke to reduce the impact of stroke progression or complication. Furthermore, the risk of recurrent stroke is highest during the first 90 days after an index stroke; longitudinal studies indicate that approximately 1 out of every 2 recurrences occurring in the first year occurs within the first 90 days^26^. Since patients with acute stroke may be admitted repeatedly for rehabilitation within 3 months after the first stroke. Factors associated with re-stroke in 1,703 patients with stroke were analyzed, including demographic characteristics, index stroke type (i.e., ischaemic or haemorrhagic), comorbidities, and COPD and allied conditions medications.

### Statistical analysis

All statistical analyses were performed using the SAS statistical package, version 9.4 (SAS, Inc.; Cary, NC, USA). Comparisons of categorical variables were performed using the χ2 test, or Fisher’s exact test if case numbers were < 5 in either cell of cross-table. Continuous variables were tested by non-parametric Kruskal–Wallis test. The stepwise Cox proportional hazards regression was performed to test the effects of all variables on stroke and was applied in the time to event model. The Kaplan-Meier method was used to estimate the risk of stroke as a function of time. All comparisons were two-sided, and *p* < 0.05 was considered statistically significant.

## Results

### Stroke incidence increased in patients with COPD and allied conditions

The study cohort comprised 24,173 patients with newly-diagnosed COPD and allied conditions. The median age was 64 years (interquartile range [IQR] = 54–73 years) and 9883 (40.9%) were female. Table 1 shows the comparisons of baseline characteristics and comorbidities between COPD and control groups. In the COPD group, 1003 (4.2%) were admitted to hospital for stroke during a median observation time of 5.3 years (IQR = 2.4–8.4 years), compared to 391 (1.6%) strokes in matched control patients during a median observation time of 5.5 years (IQR = 2.7–8.5 years). Patients with newly-diagnosed COPD and allied conditions had a significantly higher incidence of stroke (adjusted HR 2.72 with 95% CI, 2.42–3.05, *p* < 0.001) compared to the non-COPD controls (Table 2).

**Table 1:** Comparison of population with COPD and control group with respect to characteristics in demographics and comorbidities (*n* = 48,343).

Variable	COPD (*n* = 24,173)			Control (*n* = 24,170)			*P* value
	*n*	%		*n*	%		
Median age (IQR)	64 (54–73)			64 (54–73)			1
Female	9883		40.9	9882		40.9	1
Stroke	1003		4.2	391		1.6	<0.001
Re_stroke	126		0.5	13		0.1	<0.001
Comorbidity							
Diabetes mellitus	5670		23.5	7221		29.9	<0.001
Hypertension	13,291		55.0	14,881		61.6	<0.001
Dyslipidemia	8152		33.7	9410		39.0	<0.001
Atrial fibrillation	621		2.6	626		2.6	0.88
Mean observation years	5.5 ± 3.5			5.6 ± 3.4			

*IQR* interquartile range, *COPD* chronic obstructive pulmonary disease.

**Table 2:** Crude and adjusted hazard ratios for stroke among patients with COPD compared to control.

	Total	COPD *n* = 24,173	Control *n* = 24,170
Stroke	1394 (2.9%)	1003 (4.2%)	391 (1.6%)
Crude HR (95% CI)		2.62 (2.33–2.95)***	1.00
^a^Adjusted HR (95% CI)		2.72 (2.42–3.05)***	1.00

*COPD* chronic obstructive pulmonary disease, *HR* hazard ratio, *CI* confidence interval.

^a^Adjustments were made for age, sex, diabetes mellitus, hypertension, dyslipidemia, and atrial fibrillation.

*** Indicates *p* < 0.001.

**Fig. 2:**
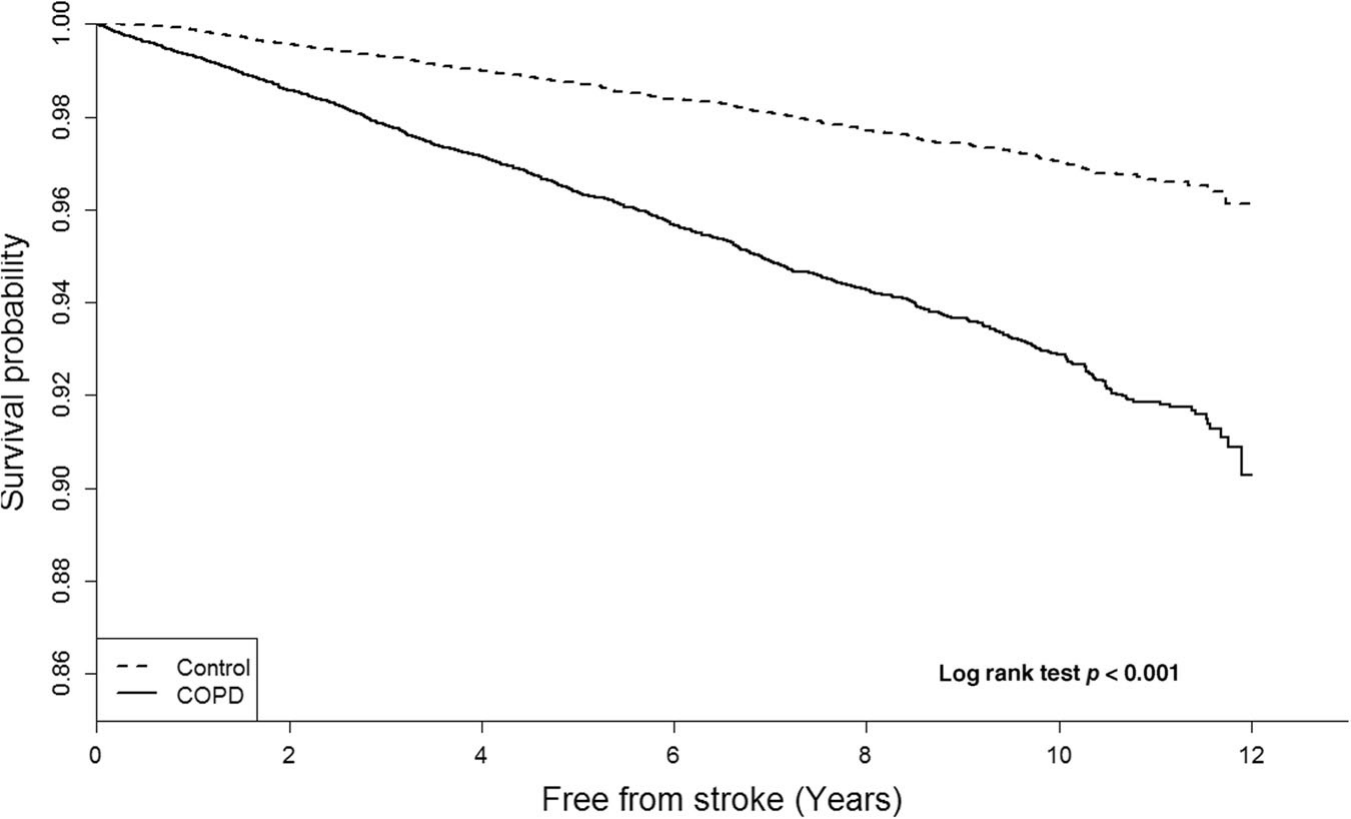
Kaplan–Meier curve plot of COPD and non-COPD control groups for stroke over time. Patients with newly-diagnosed COPD and allied conditions had a significantly higher adjusted hazard ratio (HR) of 2.75 (95% confidence interval [CI], 2.46–3.08, *p* < 0.001) for stroke with time, compared to the non-COPD control group (log rank test, *p* < 0.001).

Figure 2 illustrates the results of the Kaplan–Meier curve analysis for the COPD and control groups. Stroke incidence in COPD group increased significantly with time compared to that of the non-COPD control group (log rank test, *p* < 0.001). After adjustments made for the common stroke risk factor, including age, sex, diabetes mellitus, hypertension, dyslipidemia, and atrial fibrillation, patients with newly-diagnosed COPD and allied conditions still had a significantly higher incidence of stroke.

### Risk factors of stroke analyses in COPD and allied conditions

In the COPD group, 1003 patients (4.2%) had stroke and 23,170 patients (95.8%) had no stroke. Table 3 shows the demographic and clinical characteristics, comorbidities, and total prescription days of each class of COPD and allied conditions treatment medications. The patients with stroke were significantly older and more likely to be male than those without stroke; they also exhibited a significantly higher rate of DM, hypertension, and AF but had lower incidence of dyslipidemia.

**Table 3:** Comparison of characteristics in demographics, comorbidities, and medications among patients with and without stroke in study cohort with COPD (*n* = 24,173).

Variable	Stroke (*n* = 1003)		No stroke (*n* = 23,170)		*P* value
	*n*	%	*n*	%	
Median age (IQR)***	72 (64–79)		63 (54–73)		<0.001
Female***	326	32.5	9557	41.3	<0.001
Spirometry	410	40.9	10,200	44.0	0.05
Comorbidity					
Diabetes mellitus***	298	29.7	5372	23.2	<0.001
Hypertension***	724	72.2	12,567	54.2	<0.001
Dyslipidemia***	281	28.0	7871	34.0	<0.001
Atrial fibrillation***	66	6.6	555	2.4	<0.001
Medication					
SABA*	10	1.0	468	2.0	0.02
LABA	8	0.8	229	1.0	0.55
SAMA	34	3.4	596	2.6	0.11
LAMA*	45	4.5	1,417	6.1	0.03
Methylxanthine*	308	30.7	6378	27.5	0.03
Mucolytics***	115	11.5	4154	18.0	<0.001
ICS	24	2.4	746	3.2	0.14
ICS-LABA**	93	9.3	2869	12.4	0.003
Mean observation years***	4.0 ± 2.9		5.6 ± 3.5		<0.001

*COPD* chronic obstructive pulmonary disease, *IQR* interquartile range, *SABA* short-acting β2-agonists, *LABA* long-acting β2-agonists, *SAMA* short-acting muscarinic antagonist, *LAMA* long-acting muscarinic antagonist, *ICS* inhaled corticosteroids.

*<0.05; **<0.01; ***<0.001.

**Fig. 3:**
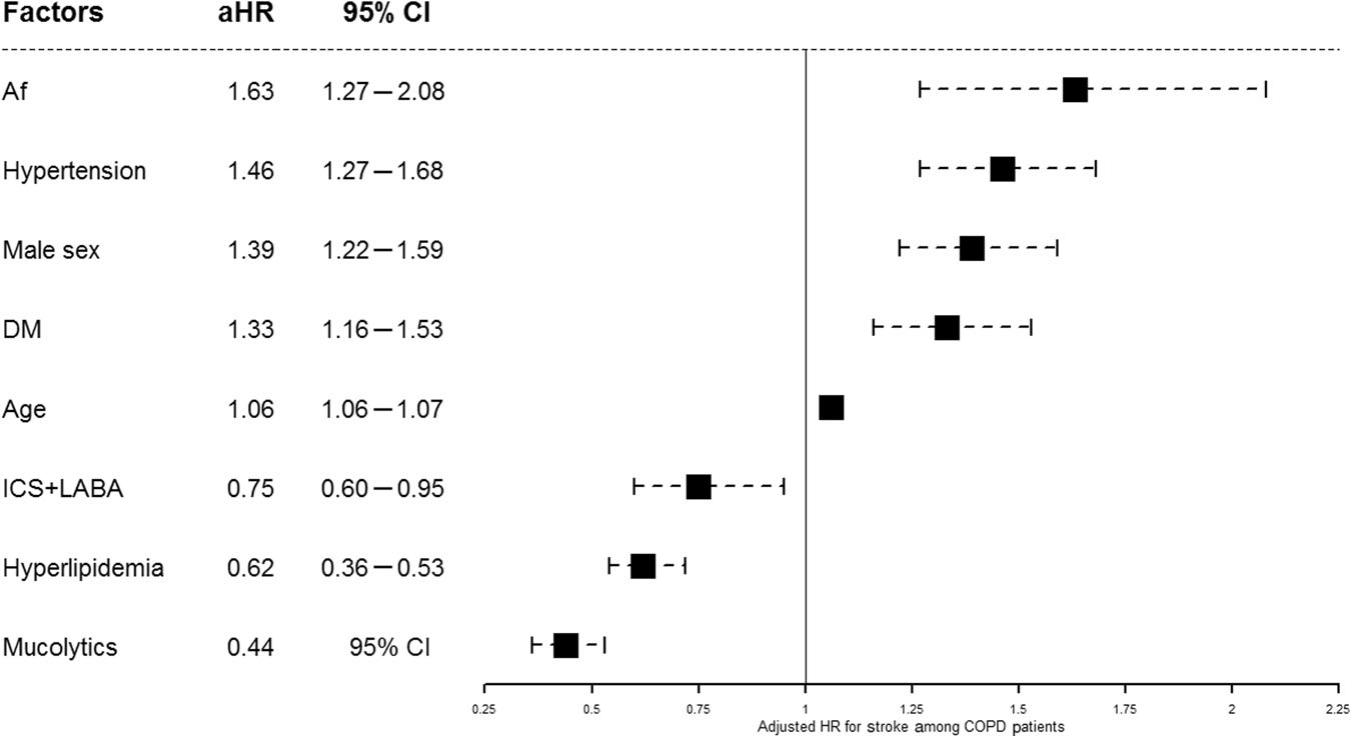
Cox proportional hazards regression in time-to-event model for stroke risk factors analysis in COPD and allied conditions patients. Multivariate analysis revealed that age (hazard ratio [HR] = 1.06, 95% confidence interval [CI]: 1.06–1.07, *p* < 0.001), male sex (HR = 1.39, 95% CI: 1.22–1.59, *p* < 0.001), hypertension (HR = 1.46, 95% CI: 1.27–1.68, *p* < 0.001), diabetes mellitus (DM) (HR = 1.46, 95% CI: 1.16–1.53, *p* < 0.001), and atrial fibrillation (AF) (HR = 1.46, 95% CI: 1.27–2.08, *p* < 0.001) significantly increased the risk of stroke among patients with COPD and allied conditions. (ICS: inhaled corticosteroid, LABA long-acting inhaled beta-agonists).

Univariate analysis revealed that SABA, LAMA, mucolytics, and agents of ICA combined with LABA were prescribed significantly less often in stroke patients than in those without stroke but more methylxanthine was prescribed in stroke patients compared to those without stroke. Table 4 shows the comparison of characteristics in demographics, comorbidities, medications between groups with or without re-stroke of patients with COPD and stroke (*n* = 1003). Figure 3 demonstrates the adjusted HR and range of 95% CI of the stroke risk factors and protective factors. It also shows the results of multivariate analysis, including that age (HR = 1.06, 95% CI: 1.06–1.07, *p* < 0.001), male sex (HR = 1.39, 95% CI: 1.22–1.59, *p* < 0.001), hypertension (HR = 1.46, 95% CI: 1.27–1.68, *p* < 0.001), DM (HR = 1.46, 95% CI: 1.16–1.53, *p* < 0.001), and AF (HR = 1.46, 95% CI: 1.27–2.08, *p* < 0.001) was associated with significantly increased risk of stroke among patients with COPD. However, dyslipidemia (HR = 0.62, 95% CI: 0.54–0.72, *p* < 0.001), mucolytics (HR = 0.44, 95% CI: 0.36–0.53, *p* < 0.001), agents with ICS-LABA combination (HR = 0.75, 95% CI: 0.60–0.95, *p* = 0.02) were associated with significant reduction in stroke risk.

**Table 4:** Comparison of characteristics in demographics, comorbidities, medications between groups with or without re-stroke of patients with COPD and stroke (*n* = 1003).

Variable	Re-stroke (*n* = 126, 12.6%)		No re-stroke (*n* = 877, 87.4%)		*P* value
	*n*	%	*n*	%	
Median age (IQR)	70 (62–77)		72 (64–79)		0.04*
Female	36	28.6	290	33.1	0.31
Stroke type, infarct	114	90.5	691	78.8	<0.01**
Comorbidity					
Diabetes mellitus	39	31.0	259	29.5	0.74
Hypertension	81	64.3	643	73.3	0.03*
Dyslipidemia	23	18.3	258	29.4	0.01*
Atrial fibrillation	11	8.7	55	6.3	0.30
Medication					
SABA	0	0	10	1.1	0.23
LABA	0	0	8	0.9	0.28
SAMA	4	3.2	30	3.4	0.89
LAMA	4	3.2	41	4.7	0.45
Methylxanthine	34	27.0	274	31.2	0.33
Mucolytics	15	11.9	100	11.4	0.87
ICS	3	2.4	21	2.4	1.00
ICS-LABA	7	5.6	86	9.8	0.14

*IQR* interquartile range.

## Discussion

In this population-based cohort study, COPD and allied conditions patients were associated with a higher risk of stroke over time than patients without COPD and allied conditions. Major comorbidities were shown to increase risk of stroke, while the use of mucolytic agents and combination ICS-LABA treatment were associated with risk reduction. Advanced age and male gender were also associated with increased stroke risk in COPD and allied conditions patients. This is the first study to raise the possibility that medications used for treating COPD and allied conditions patients may decrease stroke risk. The study only suggests potential correlation, and that needs to be further confirmed by higher-quality prospective cohort studies.

COPD and stroke are both leading causes of morbidity and mortality worldwide^27,28^. Advanced age, smoking, systemic inflammation, and oxidative stress appear to play major roles in the pathophysiological links between COPD and stroke^6,13,15,16,29^. Recent studies have demonstrated that underlying comorbidities such as cardiovascular disease (CVD) and chronic kidney disease (CKD) are independent risk factors for cardiovascular events in COPD patients^30–32^. In COPD patients with both CKD and CVD, this risk for stroke is intensified to 6.32-fold over that of those with neither of these comorbidities^30^. Results of the present study showed that COPD and allied conditions patients who were older adults, male, with hypertension, DM and atrial fibrillation had significantly higher risk of stroke. Since comorbidities are frequent in COPD and significantly affect patient’s quality of life, exacerbation frequency, and survival^1,2^, underlying comorbidities could be considered as major “treatable traits” of COPD^33–35^.

Many observational studies and meta-analyses have reported that increased cardiovascular risk in patients with COPD is associated with their use of long-acting bronchodilators^19,36–39^. Nevertheless, randomized controlled trials have failed to show an increased cardiovascular risk^40–42^. A recent study also observed that COPD patients with baseline CVD had higher risk of cardiovascular effects than those without, regardless of treatment regimens for COPD^30^. Results of the present study indicated that COPD and allied conditions patients treated with bronchodilators such as LABA and LAMA did not have increased risk of stroke, in fact, COPD and allied conditions patients treated with mucolytics or ICS-LABA actually had reduced risk of stroke. Exacerbations of COPD may also be reduced in patients treated with mucolytics^43,44^. In addition, those treated with inhaled ICS-LABA also have been shown to have reduced risk of stroke^30,45^. ICS-LABA combined treatment is shown to modulate systemic inflammation and mucolytics also may have antioxidant characteristics that could possibly alter the risk of stroke in patients with COPD.

Our findings presented that the use of LAMA in COPD cohort (4.5 and 6.1%) was lower than the use of ICS-LABA (9.3 and 12.4%). The patients in the study were from a population-based data. There was the potential risk of patient misclassification, and not all the prescriptions of inhaled bronchodilators were regulated by guidelines. Thus, the COPD treatment may not be standardized. In addition, a nationwide population-based study in Taiwan based on NHI claims data for 2009–2011 suggested that patients with asthma and COPD overlap (ACO) experience a higher disease burden than patients with asthma or COPD alone. Therefore, ICS-LABA therapy contributed the most to the total medication use, followed by LAMA monotherapy in the ACO and COPD cohort (ACO, ICS-LABA v.s. LAMA: 35.0% v.s. 9.5%; COPD, ICS-LABA v.s. LAMA: 11.1% v.s. 6.9%, respectively)^46^.

Results of the present study revealed that COPD and allied conditions patients with hyperlipidemia had a decreased risk of stroke. Hyperlipidemia, that is, high levels of total cholesterol and low-density lipoprotein (LDL) cholesterol are both associated with an increased risk of ischaemic stroke^47^. Large-scale evidence from randomized trials shows that each 1 mmol/L reduction in LDL cholesterol with statin therapy produces a proportional reduction of about 25% during each year in the rate of major vascular events^48^. Lowering LDL cholesterol by 2 mmol/L with an effective statin regimen for about 5 years in 10,000 patients would typically prevent major vascular events in about 1000 (10%) patients at high risk of heart attacks and strokes^48^. At the same time, statins were also found to reduce the level of inflammation in people with COPD, although this did not result in any clear improvement in exacerbations, mortality, functional capacity, quality of life, or lung function^49^. Results of the present study used the database released by the National Health Insurance of Taiwan, in which statins are routinely prescribed when patients are diagnosed with hyperlipidemia. Therefore, we infer that the use of statins in COPD patients with hyperlipidemia is associated with decreased risk of stroke.

The lower prevalence of DM, hypertension, and dyslipidemia in COPD population compared to control group may result from nutritional depletion and racial difference. First, COPD increases nutritional requirements, alters metabolic processes, compromises nutritional intake, and results in nutritional depletion consequently^50^. Patients with COPD for a long time may develop weight loss, sarcopenia, and pulmonary cachexia and therefore have a lower risk of metabolic syndrome. As the relative old age of our study cohort (median: 63 years), chronic undernutrition may reduce the development of these 3 comorbidities. A previous study on primary care data showed a similar reduction in DM prevalence in older COPD patients (OR for ≥75 years: 0.8 (0.7–0.9) for ex-smoker and 0.7 (0.7–0.8) for current smoker)^8^. Second, Japanese studies reported different characteristics of Japanese COPD patients from those of Westerners, including older age, lower BMI (body mass index), more emphysema-dominant lung disease, lower prevalence of cardiovascular comorbidities, DM, and metabolic syndrome^51,52^. The authors attributed these findings to the difference in COPD pathophysiology between these two populations, including ethnic/genetic, environmental, lifestyle, and socioeconomic factors. Since our study populations are also Asians, our research results are closer to the aforementioned Japanese studies.

The present study has several limitations, including that a secondary database was used and data were analyzed retrospectively, which limits the inference of causation. Smoking and BMI status are not available in the NHIRD. The influences of result were not evaluated in this study. Also, although the present study demonstrated that COPD and allied conditions patients have higher risk of stroke incidence, the percentages of major comorbidities such as hypertension, DM and atrial fibrillation were lower in COPD group compared to the non-COPD control group, which may demonstrate selection bias. Further, even though the LHID used in this study shows no significant differences in the distribution of age and sex between the subset and the original NHIRD, because this study aimed to assess the risk of stroke in newly-diagnosed COPD and allied conditions patients, we excluded those who were younger than 45 years and those who had stroke or severe cardiovascular events such as myocardial infarct, heart failure and angina before the first COPD and allied conditions diagnosis. For each patient, one control subject was randomly selected from the database by matching age, sex, and year of enrolment. Therefore, selection bias may affect the distribution of these major comorbidities between COPD and control groups. Third, other medications or therapies on treating the comorbidities or stroke were not evaluated in this study. The dosage of the medication may increase significantly in exacerbation of COPD and allied conditions patients. The more acute exacerbations, the higher frequency of medication uses. In contrast to prospective studies, these confounding factors in retrospective studies may affect the results in real world.

In conclusion, COPD and allied conditions increases the risk of stroke and re-stroke, particularly in COPD and allied conditions patients with comorbidities such as hypertension, diabetes mellitus, and atrial fibrillation. The use of mucolytic agents and combination treatment of COPD and allied conditions using ICS and LABA reduces the risk of stroke in this patient population. Further prospective studies are needed to verify whether mucolytics and ICS/LABA treatments confer protective effects against stroke in patients with COPD and allied conditions.

### Reporting summary

Further information on research design is available in the Nature Research Reporting Summary linked to this article.

## Supplementary information

REPORTING SUMMARY

## Data Availability

Supporting data is available on request.
