# Supplementary material for: The effects of medications for treating COPD and allied conditions on stroke: a population-based cohort study
Source: NPJ Prim Care Respir Med. 2022 Jan 17;32:4. doi: 10.1038/s41533-021-00267-3 (PMC8764093; doi:10.1038/s41533-021-00267-3)
Supplement: Supplementary file 1 — REPORTING SUMMARY [file 41533_2021_267_MOESM1_ESM.pdf]

nature portfolio

Corresponding author(s): NPJPCRM-01377R

Last updated by author(s): Sep 21, 2021

Reporting Summary

Nature Portfolio wishes to improve the reproducibility of the work that we publish. This form provides structure for consistency and transparency in reporting. For further information on Nature Portfolio policies, see our Editorial Policies and the Editorial Policy Checklist.

Statistics

For all statistical analyses, confirm that the following items are present in the figure legend, table legend, main text, or Methods section.

n/a	Confirmed	
☐	☒	The exact sample size (n) for each experimental group/condition, given as a discrete number and unit of measurement
☐	☒	A statement on whether measurements were taken from distinct samples or whether the same sample was measured repeatedly
☐	☒	The statistical test(s) used AND whether they are one- or two-sided<br>*Only common tests should be described solely by name; describe more complex techniques in the Methods section.*
☐	☒	A description of all covariates tested
☐	☒	A description of any assumptions or corrections, such as tests of normality and adjustment for multiple comparisons
☐	☒	A full description of the statistical parameters including central tendency (e.g. means) or other basic estimates (e.g. regression coefficient) AND variation (e.g. standard deviation) or associated estimates of uncertainty (e.g. confidence intervals)
☐	☒	For null hypothesis testing, the test statistic (e.g. F, t, r) with confidence intervals, effect sizes, degrees of freedom and P value noted<br>*Give P values as exact values whenever suitable.*
☐	☒	For Bayesian analysis, information on the choice of priors and Markov chain Monte Carlo settings
☐	☒	For hierarchical and complex designs, identification of the appropriate level for tests and full reporting of outcomes
☐	☒	Estimates of effect sizes (e.g. Cohen's d, Pearson's r), indicating how they were calculated

Our web collection on statistics for biologists contains articles on many of the points above.

Software and code

Policy information about availability of computer code

Data collection	This study extracted patient data from the Longitudinal Health Insurance Database (LHID), which is a random subset of the Taiwan National Health Insurance Research Database (NHIRD). We identified all LHID patients who were diagnosed with COPD and allied conditions (ICD-9 code 490-493 [490 Bronchitis, not specified as acute or chronic; 491 Chronic bronchitis; 492 Emphysema; 493 Asthma) by pulmonary specialists in outpatient services and hospital admission records between January 1, 2000 and December 31, 2013.
Data analysis	All statistical analyses were performed using the SAS statistical package, version 9.4 (SAS, Inc.; Cary, NC, USA). Comparisons of categorical variables were performed using the χ2 test, or Fisher's exact test if case numbers were < 5 in either cell of cross-table. Continuous variables were tested by non-parametric Kruskal-Wallis test. Stepwise logistic regression was performed to test the effects of all variables on stroke in the exposure-event model and Cox proportional hazards regression was applied in the time-to-event model. The Kaplan-Meier method was used to estimate the risk of stroke as a function of time.

For manuscripts utilizing custom algorithms or software that are central to the research but not yet described in published literature, software must be made available to editors and reviewers. We strongly encourage code deposition in a community repository (e.g. GitHub). See the Nature Portfolio guidelines for submitting code & software for further information.

Data

Policy information about availability of data

All manuscripts must include a data availability statement. This statement should provide the following information, where applicable:

- Accession codes, unique identifiers, or web links for publicly available datasets
- A description of any restrictions on data availability
- For clinical datasets or third party data, please ensure that the statement adheres to our policy

All data generated or analysed during this study are included in this published article.

Field-specific reporting

Please select the one below that is the best fit for your research. If you are not sure, read the appropriate sections before making your selection.

☒ Life sciences ☐ Behavioural & social sciences ☐ Ecological, evolutionary & environmental sciences

For a reference copy of the document with all sections, see nature.com/documents/nr-reporting-summary-flat.pdf

Life sciences study design

All studies must disclose on these points even when the disclosure is negative.

Sample size	The population-based study cohort comprised 24,173 patients diagnosed with COPD and allied conditions between 2000 and 2013, and 24,170 selected matched patients without COPD comprised the comparison cohort from a nationwide database.
Data exclusions	Patients with any record of having COPD and allied conditions before 2002 (n = 9,432) were excluded. We excluded code 494-496 patients with lung disease accompanied by COPD (494 Bronchiectasis; 495 Extrinsic allergic alveolitis; 496 Chronic airway obstruction, not elsewhere classified) (n = 2080). We also excluded those who were younger than 45 years (n = 8,200) and those who had stroke (ICD-9 code 430-434, 436, 438), myocardial infarct (code 410), heart failure (code 428), angina (code 413), tuberculosis (code 010.90), or lung cancer (code 162) before the first COPD diagnosis (n = 8,506). Patients who had no further follow-up in their medical records after admission for COPD and allied conditions, which may be suggestive of death, were also excluded (n = 221).
Replication	The study findings are not replicated and can not be reproduced.
Randomization	To construct the control group, for each included patient, one non-COPD and allied conditions patient was randomly selected from the database by matching age, sex, and year of enrollment. The same exclusion criteria for the COPD and allied conditions group were applied to selection of controls. Three patients had no matched control, therefore, the control group included only 24,170 subjects.
Blinding	For the studied cases, the index date was defined as the earliest date of COPD and allied conditions diagnosis. For the control group without COPD and allied conditions, the first outpatient department visit date in the comparison year was assigned as the index date.

Reporting for specific materials, systems and methods

We require information from authors about some types of materials, experimental systems and methods used in many studies. Here, indicate whether each material, system or method listed is relevant to your study. If you are not sure if a list item applies to your research, read the appropriate section before selecting a response.

Materials & experimental systems

n/a	Involved in the study	
☒	☐	Antibodies
☒	☐	Eukaryotic cell lines
☒	☐	Palaeontology and archaeology
☒	☐	Animals and other organisms
☒	☐	Human research participants
☐	☒	Clinical data
☒	☐	Dual use research of concern

Methods

n/a	Involved in the study	
☒	☐	ChIP-seq
☒	☐	Flow cytometry
☒	☐	MRI-based neuroimaging

Clinical data

Policy information about clinical studies

All manuscripts should comply with the ICMJE guidelines for publication of clinical research and a completed CONSORT checklist must be included with all submissions.

Clinical trial registration	N/A
Study protocol	N/A
Data collection	N/A
Outcomes	N/A
